# High-Resolution Melting Analysis Potential for *Saccharomyces cerevisiae* var. *boulardii* Authentication in Probiotic-Enriched Food Matrices

**DOI:** 10.3390/biotech13040048

**Published:** 2024-11-14

**Authors:** Monika Borkowska, Michał Kułakowski, Kamila Myszka

**Affiliations:** Department of Biotechnology and Food Microbiology, Poznan University of Life Sciences, Wojska Polskiego 48, 60-637 Poznan, Polandkamila.myszka@up.poznan.pl (K.M.)

**Keywords:** *Saccharomyces cerevisiae* var. *boulardii*, identification, probiotic, dietary supplement, probiotic-enriched food, qPCR-HRM

## Abstract

To date, the only probiotic yeast with evidence of health-promoting effects is *Saccharomyces cerevisiae* var. *boulardii*. The expanded market including dietary supplements and functional foods supplemented with *Saccharomyces cerevisiae* var. *boulardii* creates an environment conductive to food adulterations, necessitating rapid testing to verify product probiotic status. Herein, qPCR-HRM analysis was tested for probiotic yeast identification. The effectiveness of the primer pairs’ set was examined, designed to amplify heterogeneous regions in (a) rDNA sequences previously designed to identify food-derived yeast and (b) genes associated with physiological and genotypic divergence of *Saccharomyces cerevisiae* var. *boulardii.* Preliminary tests of amplicons’ differentiation power enabled the selection of interspecies sequences for *18SrRNA* and ITS and genus-specific sequences *HO*, *RPB2*, *HXT9* and *MAL11.* The multi-fragment qPCR-HRM analysis was sufficient for culture-dependent *Saccharomyces cerevisiae* var. *boulardii* identification and proved effective in the authentication of dietary supplements’ probiotic composition. The identification of *S. cerevisiae* var. *boulardii* in complex microbial mixtures of kefir succeeded with more specific intragenus sequences *HO* and *RPB2.* The predominance of *S. cerevisiae* var. *boulardii* in the tested matrices, quantitatively corresponded to the probiotic-enriched food, was crucial for identification with qPCR–HRM analysis. Considering the reported assumptions, qPCR-HRM analysis is an appropriate tool for verifying probiotic-enriched food.

## 1. Introduction

Health-promoting effects were predominantly demonstrated for specific probiotic strains of the bacteria genera *Lactobacillus* and *Bifidobacterium* [1,2], while the only yeast with a probiotic status evidenced clinically is *Saccharomyces cerevisiae* var. *boulardii* (*Sb*) [3,4,5]. The clinical studies did not show any major side effects related to *Sb*. However, some cases of *Sb* fungemia have been documented [6,7,8,9,10], prompting a search for methods of *Saccharomyces cerevisiae* (*Sc*) intraspecies differentiation.

The taxonomic position of *Sb* has been controversial over the years [4,11,12,13,14,15]. McFarland indicated some crucial divergences at the physiological (i.e., lack of ability to use galactose as carbon source and lack of ability to produce ascospores) and molecular levels (i.e., individual chromosome and gene copy numbers) between *Sb* and *Sc* [16]. Using comparative genomic hybridization for whole-genome analysis, *Sb*’s physiological and genotypic distinctive features were confirmed [17]. The observations concerned the specific properties of Ty elements (yeast retrotransposons) and the copy number of genes in its subtelomeric regions. These were confirmed in the subsequent genomic comparative study, which additionally showed that *Sb* strains are closely related to *Sc* wine strains [18]. Pais and colleagues hypothesized that different phenotypes exhibited by *Sb* and *Sc* might result from variations in gene expression control. They demonstrated that *Sb* did not share conserved promoter regions and transcription factor binding sites with *Sc* [19]. 

So far, the search for methods of intraspecies differentiation of *Sc* has mainly involved clinical studies. By combining randomly amplified polymorphic DNA-PCR, restriction fragment length polymorphism analysis of rDNA spacer regions and pulsed-field gel electrophoresis, all examined *Sc* isolates were discriminated in clinical research. Moreover, probiotic Sb strains were demonstrated to form a separate cluster within the species [20]. Additionally, for intraspecies differentiation of probiotic and clinical *Sc* strains, a powerful microsatellite-based technique was developed [21]. Furthermore, a high level of discrimination was achieved by hybridization with retrotransposon Ty917 [22]. Finally, a rapid multiplex PCR method unequivocally identified probiotic-derived *Sc* isolates [23].

High-Resolution Melting (HRM) analysis is a state-of-the-art technique that enables the differentiation at the single base resolution of DNA fragments amplified in qPCR. It is an alternative single-tube approach with no time-consuming post-PCR processing or the need for sequencing to detect DNA polymorphisms. HRM analysis was initially used in clinical research and diagnostics, but its robust potential and technological advancements enable it to expand into other areas of the life sciences. In food sciences, HRM analysis has already been used for the differentiation of food-derived yeast species including *Saccharomyces* spp. [24], sourdough yeast [25,26] and spoilage yeast [27,28,29]. All procedures presented a high potential for HRM analysis in yeast species differentiation based on the amplification of regions within rRNA genes or ITS non-coding sequences characterized by low intraspecific variability and high interspecific polymorphism [30,31]. The vast majority of the studies cited dealt with culture-dependent identification. The culture-independent identification of sourdough yeast by HRM analysis did not yield conclusive results [25,26]. 

This paper presents pioneering research focused on the development of an efficient tool for the rapid identification of *Sb*, not only in pure culture but especially in microbiologically complex food matrices. The efficiency of previously designed primer pairs for rDNA sequences and newly presented intragenus primer pairs associated with physiological and genotypic characteristics of *Sb* were verified in the promising qPCR–HRM analysis. The utility of selected primer pairs was verified in dietary supplements presenting the single yeast composition. The current state of the regulations and the expanded supplement market create an environment conducive to food adulterations, necessitating rapid testing to verify product status. The problem may soon affect a much wider range of foods. Concerning probiotic yeast, intense research activity arose in developing functional foods supplemented with *Sb* not only as a probiotic agent, but also as a key element for the generation of bioactives, increasing the antioxidant capacity [32,33], improving nutritional value [34], or for the stabilization of LAB strains throughout fermentation and storage [35,36]. Therefore, the effectiveness of the designed analysis was also checked for multi-yeast mixtures corresponding in composition to kefir, a natural source of probiotic yeast origin [37,38].

## 2. Materials and Methods

### 2.1. Biological Material

#### 2.1.1. Strains

The following reference strains were used in the optimization of qPCR–HRM analysis focused on identifying probiotic strains of *Saccharomyces cerevisiae*: (i) *Saccharomyces boulardii* CNCM I-745 (Enterol, Biocodex, Gentilly, France) (*Sb*_745_) and *Saccharomyces boulardii* CNCM-I-3799 (Oslonik max extra, TZF Polfa, Warsaw, Poland) (*Sb*_3799_) isolated from probiotic preparations under this project; (ii) and collection strains *Saccharomyces cerevisiae* ATCC 9763 (*Sc*_ATCC9763_) and *Saccharomyces cerevisiae* Ethanol Red (Lesaffre; Marcq-en-Baroeul, France) (*Sc*_EtRed_). Additionally, (i) *Kluyveromyces marxianus* DSM 5422 (German Collection of Microorganisms and Cell Cultures GmbH; Braunschweig, Germany) (*Km*), (ii) food-derived isolates *Saccharomyces cerevisiae* (*Sc*_D_) and *Pichia fermentans* (*Pf*_D_) deposited in the microbial collection of Department Biotechnology and Food Microbiology (DBFM) in Poznan University of Life Sciences (PULS) and (iii) *Lactobacillus delbrueckii* subsp. *lactis* DSM 20072 (German Collection of Microorganisms and Cell Cultures GmbH; Germany) (*Ld*sub*l*) were used in the subsequent stages of testing the qPCR–HRM. All strains were kept as glycerol stocks at −80 °C. Yeast strains were recovered on YPD agar plates [(g L^−1^): yeast extract, 10 (Biomaxima, Lublin, Poland); bactopeptone, 20 (BTL, Łódź, Poland); glucose, 20 (POCh, Gliwice, Poland); and agar, 15 (BTL)] and lactic acid bacteria (LAB) were retrieved with De Man, Rogosa and Sharpe (M.R.S) agar (BTL).

#### 2.1.2. Probiotic Supplements

Four probiotic preparations classified as dietary supplements and available in local pharmacies were analyzed with the optimized qPCR–HRM method. The composition of the chosen products is shown in Table 1.

### 2.2. Microbiological Methods

#### 2.2.1. *S. boulardii* Reference Strains

The reference strains of *Sb* used in this study were isolated from two different commercial products, one with proven therapeutic effects and the other classified as a dietary supplement. To revive the yeast, the contents of each capsule (250 mg) were suspended in 20 mL of YPD in a sterile 50 mL Falcon tube. The mixture was then shaken at 150 rpm at 30 °C overnight. Afterwards, the log serial dilutions were made from the overnight culture. 100 µL of each dilution was transferred in duplicate onto Yeast extract Glucose Chloramphenicol agar (YGC) (BTL, Łódź, Poland). Incubation was carried out overnight at 30 °C. Single colonies were then reinoculated onto fresh YGC agar. The yeast isolates were first verified based on morphological characteristics with microscopy and then subjected to MALDI TOF mass spectrometry identification.

#### 2.2.2. Sporulation Test

##### Procedure of Sporulation Induction

For determination of the ascospore formation, 5 mL of YPD medium in a 50 mL Falcon tube was inoculated with a fresh yeast colony. Each culture was incubated at 30 °C in a shaking incubator at 150 rpm for 18–20 h. 200 µL of the overnight culture was transferred into 5 mL of liquid YPD and incubated at 30 °C in a shaking incubator until the cell suspension reached OD equal to 1. Then the culture was centrifuged at 1811× *g* for 5 min and the supernatant was discarded. The pellet was resuspended in 5 mL of pre-sporulation medium (g L^−1^: yeast extract, 10; pepton, 20; potassium acetate, 10 (POCh, Gliwice, Poland)) and grew for 18–24 h at 30 °C in shacking incubator. The culture was centrifuged at 1811× *g* for 5 min and the supernatant was discarded. Finally, the pellet was resuspended in 5 mL of sporulation medium (g L^−1^: potassium acetate, 10) and allowed to sporulate for 48 h at 30 °C in a shaking incubator. The tested strains were subjected to the sporulation procedure in two independent replicates.

##### Cells’ Ziehl-Neelsen Staining

The basic dye, concentrated carbol fuchsin (g L^−1^: fuchsin, 33.3 (Chempur, Piekary Śląskie, Poland), phenol, 66.7 (Chempur) and 167 mL of ethanol (POCh, Gliwice, Poland)), was applied to the fixed smear of yeast on a degreased basic slide for 15 min. During staining the slide was heated with a burner (to the so-called “three pairs”). Then the preparation was discolored in a 3% solution of hydrochloric acid (Honeywell, Charlotte, NC, USA) in ethanol (POCh, Gliwice, Poland) (acid alcohol). Afterwards, the contrast dye 0.1% (*w*/*v*) methylene blue (Chempur) was applied for 10 min. Finally, the preparation was observed in the light microscope Primo star (Zeiss, Oberkochen, Germany) under 1000× magnification using immersion.

#### 2.2.3. The Mixtures of Lactic Acid Bacteria and Yeast Cells

A measurement of 5 mL of fresh M.R.S Broth or liquid YPD in a 50 mL Falcon tube was inoculated with a bacterial (*Ld*sub*l*) or yeast colony (*Sc*_D_, *Sb*_745_, *Km*, *Pf*_D_), respectively. The cultures were incubated at 30 °C with 250 rpm shaking for 20 h. The average cells’ concentration of each overnight culture was determined using CellDrop FL (DeNovix INC, Wilmington, NC, USA). Subsequently, mixtures of microorganisms were prepared in saline water (0.85% *w*/*v* NaCl). Suspensions included *Sc*_D_ and *Sb*_745_ cells, where *Sb* accounted for 10% (Mx_0.1), 50% (Mx_0.5) and 90% (Mx_0.9) of the total amount of yeast cells. Another group of suspensions consisted of those that contained a fixed number of *Ld*sub*l* cells and yeast cells with a 60% share of *Sc*_D_ (Mx_*Sc*) or *Sb* (Mx_*Sb*), 38% of *Km* and 2% of *Pf*_D_. In this series of mixtures, the ratio of *Sc* cells to *Sb* cells was the same as in suspensions composed only of *Sc* cells (Figure S1). 

### 2.3. MALDI-TOF Mass Spectrometry

MALDI-TOF mass spectrometry analysis was performed in the Microbiological Laboratory of the Jagiellonian Center of Innovation (Cracow, Poland). MALDI-TOF mass spectrometer Microflex LT (Bruker Daltonics, Bremen, Germany) was applied for matrix-assisted laser desorption/ionization with time-of-flight analysis. The identification of microorganisms was based on the specific profile of ribosomal proteins compared to a representative database of bacteria, yeast-like fungi, filamentous fungi and dermatophytes, utilizing the MALDI Biotyper system (Bruker Daltonics). The level of the identification confidence was indicated by the identification indicator (*Ii*). Values of *Ii* greater or equal to 2.00 indicate high-confidence identification. Values within the range of 1.70 to 1.99 indicate low-confidence identification, while values below 1.69 indicate that no species identification is possible.

### 2.4. Total Genomic DNA Extraction Procedures

#### 2.4.1. Total Genomic DNA Isolation from Yeast Culture

Preceding DNA extraction, the studied yeast strains were freshly cultured on YPD agar at 30 °C for 20 h. DNA was extracted using the Genomic mini AX yeast spin kit (A&A Biotechnology, Gdynia, Poland), following the manufacturer’s protocol. The procedure included a step of yeast lysis with lyticase at 30 °C combined with vigorous vortexing. Extracted DNA quality was confirmed by applying agarose gel electrophoresis, following a standard method [39]. Exclusively DNA samples with sharp and intense bands were used in qPCR. DNA concentration and purity were assessed using a UV spectrophotometer, NanoDrop ND-1000 (Thermo Fisher Scientific, Wilmington, NC, USA). The OD 260/280 ratio of the DNA samples for amplification ranged between 1.8 and 2.0. The extracted DNA was stored at −20 °C.

#### 2.4.2. Total Genomic DNA Isolation from Dietary Supplements and Microbial Mixtures

The capsule contents of each dietary supplement were crushed in liquid nitrogen in a mortar. The fresh yeast and bacterial cultures were used for preparing mixtures (Section 2.2.3). Following this, 2 mL of each mixture was pelleted by centrifugation. Total genomic DNA was extracted from 100 mg of each food supplement and each mixture pellet using Genomic mini AX Food kit (A&A Biotechnology, Gdynia, Poland). The procedure was conducted according to the manufacturer’s protocol. The first step was lysis with proteinase K at 30 °C, followed by vigorous vortexing. DNA was isolated and purified from the lysate with column work through gravity and then eluted, precipitated and dissolved in sterile water. The concentration and purity of the isolated DNA were determined using a UV spectrophotometer NanoDrop ND-1000 (Thermo Fisher Scientific, Wilmington, NC, USA). The quality and integrity of the DNA samples were verified through agarose gel electrophoresis according to a standard method [39]. The extracted DNA was stored at −20 °C.

### 2.5. Quantitative Real-Time PCR—High-Resolution Melting Analysis (qPCR–HRM)

#### 2.5.1. Applied Primer Pairs

rDNA sequences (*18S rRNA*, *26S rRNA* and ITS region) of the studied yeast strains were amplified with primer pairs designed previously [40]. As part of this work, additional primer pairs were designed. *S. cerevisiae* S288C genes’ sequences were downloaded from the NCBI database. The accession numbers of sequences are shown in Table 2. The multiple sequence alignment tool from NCBI was applied to align and compare the targeted sequences to *Saccharomyces* spp. The sequence alignment was analyzed for DNA regions showing intragroup heterogeneity, flanked by conservative sequence segments enabling the attachment of primers. Amplicon length was kept within the range of 100 to 250 bp. The primers were designed with the Primer3-BLAST tool and synthesized by Merck KGaA (Darmstadt, Germany). Initially, annealing temperature optimization of all primer pairs was performed using Perpetual OptiTaq PCR MasterMix according to the manufacturer’s instructions (EURx, Gdansk, Poland) in a temperature gradient of 58–63 °C. The amplification efficiency was verified through agarose gel electrophoresis according to a standard method [39]. *TDA8* primer pair was eliminated from the further study due to a lack of amplification on *Sc* samples in the optimization step. Furthermore, amplification products of all studied primer pairs were positively verified for expected lengths (Figure S2).

#### 2.5.2. qPCR Protocol

PCR mixtures contained 5 µL of commercial qPCR master mix with Eve Green dye (Bio-Rad Laboratories, Inc., Hercules, CA, USA), 0,5 µL of 10 µM forward and reverse primer and 1–4 µL of DNA sample (5 ng of single-yeast DNA or 20 ng of microbial-mix DNA per reaction) in a total volume of 10 µL. All reactions were performed in clear-walled PCR 96-well plates using the CFX96 cycler (Bio-Rad Laboratories, Hercules, CA, USA). The amplification conditions were as follows: 95 °C for 3 min was followed by 30–40 cycles of denaturation at 95 °C for 15 s, annealing at 60 °C (primer pairs for sequence segment within *18SrRNA*, *26SrRNA*, ITS, *TEF1alpha*, *HO*, *RPB2*, *MAL11* and *HXT9*) or 58 °C (primer pairs for *CCA1*) for 30 s and extension at 72 °C for 30 s. Melting curves data were collected over a temperature range between 65 °C and 95 °C. Data acquisition was conducted in increments of 0.2 °C, with each step lasting 10 s. All DNA samples were analyzed in technical duplicates in at least two independent runs. To check the purity of reagents, No Template Control (NTC) for each primer pair was run in parallel.

#### 2.5.3. HRM Analysis

Precision Melt Analysis Software (PMAS) (Bio-Rad Laboratories, Inc.) was used to analyze the generated qPCR amplicons. The pre- and post-melt regions, as well as the intersection points of the melt curves in a temperature-shifted view, were carefully evaluated. Adjustments were made for each amplicon as necessary. The clustering settings were adjusted to improve the sensitivity detection of the melt curve shape change. A melting temperature (Tm) threshold difference of 0.2 °C was set for all melt curve analyses, and PMAS automatically assigned clustering confidence scores.

### 2.6. Statistical Analysis

The statistical significance of the differences observed based on clustering in HRM analysis of yeast strains for the four examined regions (*18SrRNA*, *26SrRNA*, ITS, *TEF1alpha*) was assessed using DarWin software version 6.0.21. The phylogenetic tree was constructed based on the unweighted pair-group method with the arithmetic averages (UPGMA).

## 3. Results

### 3.1. Characteristic of Saccharomyces cerevisiae var. boulardii Reference Strains

**Figure 1:**
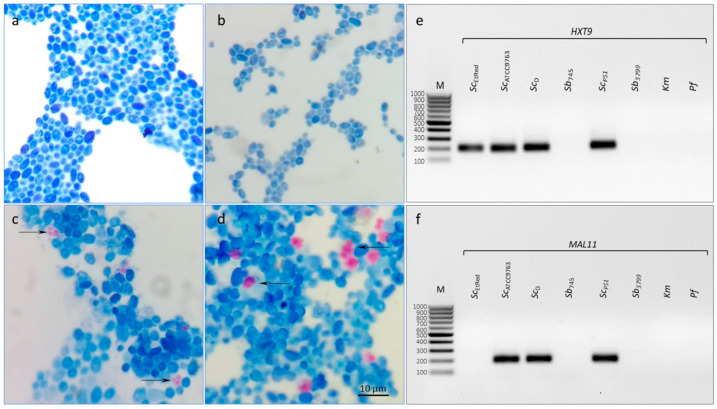
Verification of sporulation and HXT9 or MAL11 presence in Sc strains. Microscopic images of yeast cells of Sb745 (**a**), Sb3799 (**b**), ScPS1 (**c**), ScD (**d**) strains. The cells’ suspensions were induced to sporulate under starvation conditions and then stained with the Ziehl–Neelsen method. The arrows indicate stained ascospores (**c**,**d**). Electrophoretic separation of HXT9 (**e**) and MALL1 (**f**) amplicons obtained in PCR for *S. cerevisiae* var. *boulardii* reference strains (Sb745, Sb3799), *S. cerevisiae* strains (ScATCC9763, ScEtRed, ScD and ScPS1), *K. marxianus* (*Km*) and *P. fermentans* (*Pf*). M—DNA Marker 100 bp LOAD (Syngen Biotech, Wroclaw, Poland).

Two reference strains were confirmed with high confidence by MALDI-TOF mass spectrometry to belong to genus *Saccharomyces cerevisiae. Ii* was 2.16 and 2.09 for *Sb*_745_ and *Sb*_3799_, respectively. The reference strains did not show the ability to sporulate (Figure 1a,b), and no amplification of regions *MAL11* and *HXT9* was detected for either (Figure 1e,f).

### 3.2. Differentiation of Saccharomyces cerevisiae Strains Using Interspecies Primer Pairs in qPCR-HRM Analysis

**Figure 2:**
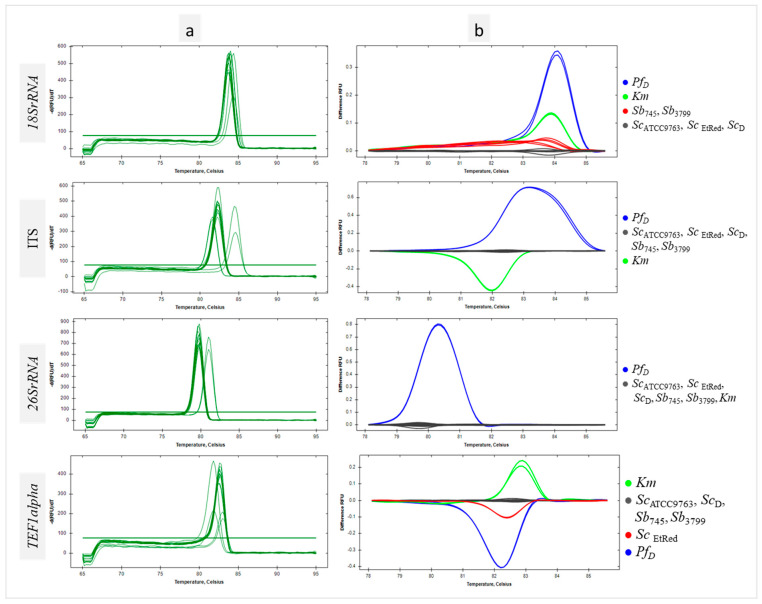
Differentiation of yeast with interspecies primer pairs in qPCR-HRM analysis. Melt peaks (**a**) and difference curves grouped as color-marked clusters (**b**) were obtained by qPCR-HRM analysis of 18SrDNA, ITS, 26SrDNA and TEF1alpha regions. DNA templates of *S. cerevisiae* var. *boulardii* reference strains (Sb745, Sb3799), *S. cerevisiae* strains (ScATCC9763, ScEtRed, ScD), *K. marxianus* (Km) and *P. fermentans* (PfD) strains were amplified in technical duplicate.

For all sequences tested, a homogeneous product was obtained for each sample as evidenced by single peaks (Figure 2a). HRM analysis of the *18SrRNA* region amplicon showed individual clustering of *Sc* and probiotic *Sb* strains at the differentiation thresholds used. The other species used in the study, the *Km* collection strain and the *Pf*_D_ isolate, were grouped separately (Figure 2b, *18SrRNA*). The maximum RFU difference between the *Sb* and *Sc* clusters was 0.03, a slightly larger difference of 0.1 was between the *Sb* and *Sc* clusters combined and the *Pf*_D_ cluster, and the largest difference of 0.3 was between the *Sb* and *Sc* clusters combined and the *Km* cluster. For the non-coding ITS sequence, all strains belonging to *Sc* species, including the probiotic ones, were in one cluster. *Km* and *Pf*_D_ control strains formed separate clusters (Figure 2b, ITS). The maximum RFU difference was 0.6 between the *Sb*/*Sc* cluster and *Km*. Slightly less difference, 0.4, was shown by comparing the *Sb*/*Sc* and *Pf*_D_ clusters (Figure 2b, ITS). According to HRM analysis, the qPCR reaction with the primer pair for the *26SrRNA* region yielded a uniform product for all *Sc* strains as well as the *Km* strain. In this case, only *Pf*_D_ was grouped separately (Figure 2b, *26SrRNA*). Amplification of a region selected within *TEF1alpha* resulted in a product whose denaturation profile was the same for all *Sc* and *Sb* tested except *Sc_EtRed_*. The *TEF1alpha* region products obtained for the negative control strains were clustered individually (Figure 2b, *TEF1alpha*). The melting temperatures of the amplicons of each of the genomic DNA regions tested did not differ within the *Sc* species significantly (Table S1). HRM-based clustering of the selected sequences analyzed with DarWin software showed that the reference probiotic strains tested are genetically very similar and phylogenetically a variant of the species *Sc* (Figure S3). 

### 3.3. Differentiation of Saccharomyces cerevisiae Strains Using Intragenus Primer Pairs in qPCR-HRM Analysis

**Figure 3:**
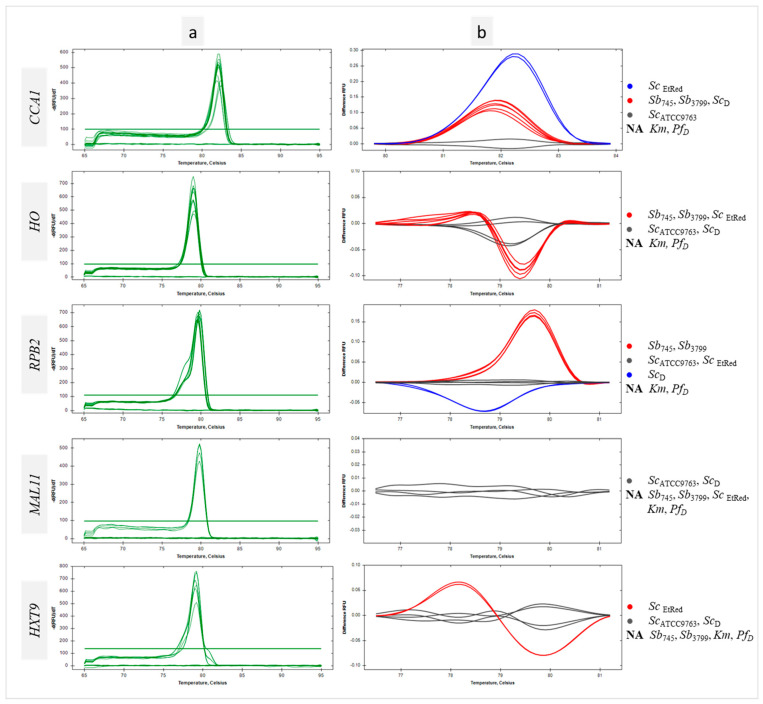
Differentiation of Sc strains using intragenus primer pairs in qPCR-HRM analysis. Melt peaks (**a**) and difference curves grouped as color-marked clusters (**b**) were obtained by qPCR-HRM analysis of CCA1, HO, RPB2, MAL11and HXT9 regions. DNA templates of *S. cerevisiae* var. *boulardii* reference strains (Sb745, Sb3799), *S. cerevisiae* strains (ScATCC9763, ScEtRed, ScD), *K. marxianus* (Km) and *P. fermentans* (PfD) strains were amplified in technical duplicate. NA—not amplified.

No products were demonstrated for *Km* and *Pf*_D_ control templates using primer pairs designed for the genus *Saccharomyces* (Figure 3 and Figure S2). Furthermore, no amplification of the *MAL11* and *HXT9* regions was observed for the reference strains *Sb* (Figure 3, *MAL11*, *HXT9*, Figure 1e,f). In addition, no sequence amplification in the *MAL11* region was detected for the *Sc_EtRed_* template (Figure 3, *MAL11*, Figure S2). For all sequences tested, a homogeneous product was obtained for the remaining samples, as evidenced by single peaks (Figure 3a). The peak melting curves of *RPB2* amplicons for *Sc*_D_ had shoulders, indicative of polymorphisms in amplified sequence. HRM software grouped *Sb* probiotic strains and *Sc_D_* isolate in one cluster at *CCA1* region analysis (Figure 3, *CCA1*). A similar result was the joint clustering of probiotic strains and *Sc_EtRed_* regarding the melting temperature and profile of *HO* sequence (Figure 3, *HO*). The maximum RFU difference in the *HO* melting profile was 0.1 between the *Sb*_745_ cluster and the *Sc*_ATCC9763_ cluster (Figure 3b, *HO*). The amplicon obtained with the primer pair designed for *RPB2* was essential in the differentiation of the probiotic strains against the other *Sc* tested in this study. The RFU difference between the clusters was 0.2 (Figure 3b, *RPB2*). Significant differences were found in the melting temperatures of *CCA1* PCR products for all *Sc* strains tested, including the two probiotic strains. Significantly, the melting temperature of *RPB2* amplicon was the same for both *Sb* strains and differed from the others by 0.2 °C (Table S1).

### 3.4. Identification of Probiotic Yeast in Dietary Supplements with qPCR-HRM Based on a Verified Set of Primer Pairs

**Figure 4:**
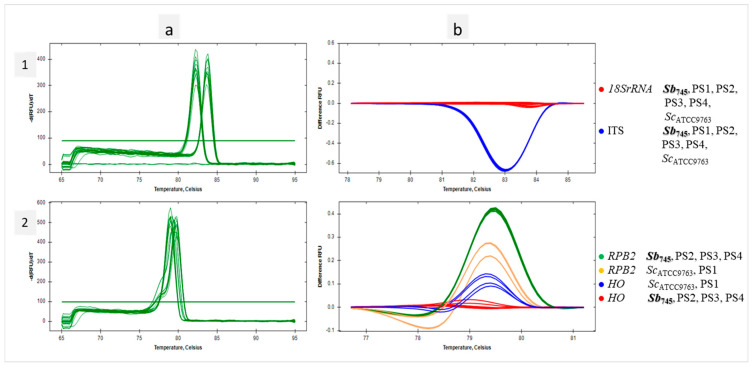
Identification of Sb in dietary supplements with qPCR-HRM. Melt peaks (**a**) and difference curves grouped as color-marked clusters (**b**) were obtained by qPCR-HRM analysis of 18SrDNA and ITS regions (1), HO and RPB2 regions (2). DNA template of *S. cerevisiae* var. *boulardii* reference strain (Sb745) as positive control, *S. cerevisiae* reference (ScATCC9763) and the dietary supplements’ DNA templates (PS1, PS2, PS3, PS4) were amplified in technical duplicate.

In the present study, a genetic analysis of four probiotic supplements available in pharmacies was performed. Their composition is presented in Table 1. DNA extracts obtained from the preparations along with reference templates of *Sb_745_* and *Sc*_ATCC9763_ were subjected to the qPCR reactions with the selected primer pairs. According to HRM analysis, the *18SrRNA* sequences for each of the test samples were grouped with the references. The same effect was observed for the amplicons of the ITS region (Figure 4(1a,b)). If *Saccharomyces*-specific primer pairs were used, only PS2, PS3 and PS4 samples were found to cluster with the *Sb_745_* reference for *HO* sequences, as well as *RPB2*. The PS1 sample remained in separate clusters for both studied sequences with *Sc*_ATCC9763_ amplicons (Figure 4(2a,b)). PS1 strain was identified by MALDI-TOF mass spectrometry as *Saccharomyces cerevisiae* with high confidence (*Ii* was 2.15). The amplification of *MAL11* and *HXT9* regions on the DNA template of the PS1 supplement was found (Figure 1e,f). In addition, the isolated strain of the PS1 supplement was able to sporulate (Figure 1c).

### 3.5. Identification of Probiotic Yeasts in Microbial Mixtures with qPCR-Based HRM Analysis 

**Figure 5:**
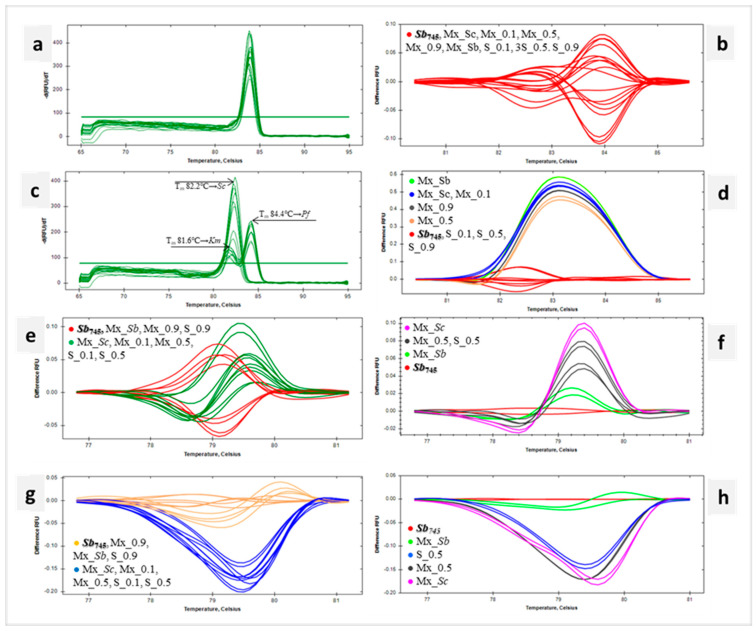
Identification of Sb745 in microbial mixtures with qPCR-based HRM analysis. Melt peaks (**a**) and difference curves grouped as color-marked clusters (**b**) were obtained by qPCR-HRM analysis of 18SrDNA. Melt peaks (**c**)—the arrows indicate species-specific peaks at T_m_ 81.6 °C, 82.2 °C and 84.4 °C for Km, ScD and PfD, respectively, and difference curves (**d**) grouped as color-marked clusters detected for ITS amplicon. Difference curves grouped as color-marked clusters obtained by qPCR-HRM analysis of HO sequence presented for all samples (**e**) and selected samples (**f**), and RPB2 sequence presented for all samples (**g**) and selected samples (**h**). DNA templates of *S. cerevisiae* var. *boulardii* reference strain (Sb745) as positive control and the microbial mixtures’ DNA extracts (Mx_Sb, Mx_0.9, Mx_0.5, Mx_0.1, Mx_Sc, S_0.9, S_0.5, S_0.1) were amplified in technical duplicate.

The mixed suspensions (Mx) for DNA isolation containing a fixed amount of *Ld*sub*l* (log 8 CFU mL^−1^), *Km* (3.8 log 6 CFU mL^−1^) and *Pf_D_* (2 log 5 CFU mL^−1^) cells were prepared. Successively, the suspensions Mx_Sc, Mx_0.1, Mx_0.5, Mx_0.9 and Mx_Sb contained increasing numbers of *Sb_745_* cells. In addition, suspensions S_0.1, S_0.5 and S_0.9 were prepared, containing only *Sc_D_* and *Sb_745_* cells, which were combined in analogous ratios to suspensions Mx (Figure S1). DNA was extracted from the obtained cells’ pellets with a kit designed for foods. The resulting templates and *Sb_745_* positive control DNA were amplified using the primer pairs for *18SrRNA*, ITS, *HO* and *RPB2* regions. Considering the *18SrRNA* sequence, the melting temperatures (T_m_) of the amplicons obtained for the following samples were in the range of 83.80–84.0 °C (Figure 5a). In the same range remained the T_m_ of the *18SrRNA* amplification products of the yeast species studied, namely *Sc*_D_, *Km* and *Pf*_D_ (Table S1). The software grouped all tested samples into one cluster (Figure 5b). More clusters were obtained in HRM analysis of ITS sequences (Figure 5d). The first grouped the reference strain *Sb*_745_ and mixtures with *Sb*_745_ and *Sc*_D_ (S_0.1, S_0.5 i S_0.9). In this cluster were amplicons with a T_m_ of 82.2 °C (Figure 5c), which corresponded to the melting point of the ITS product for *Sc*_D_ (Table S1). The remaining clusters included samples containing *Km*, *Pf*_D_, *Sc*_D_ and *Sb*_745_ in different proportions (Figure 5d). These amplification products showed double peaks with T_m_ of 81.6 °C and 84.4 °C. (Figure 5c). The T_m_ indicated in the figure corresponded to the melting temperatures of ITS sequences of *Km* and *Pf*_D_ species, respectively (Table S1). In each case, the product with a melting point corresponding to *Pf*_D_ was quantitatively predominant. Exploring the *HO* sequence, the Mx_Sb, Mx_0.9 and S_0.9 samples were clustered with the positive control in the HRM analysis. The remaining Mx_Sc, Mx_0.1, Mx_0.5, S_0.1 and S_0.5 were grouped in a separate cluster (Figure 5e). The same result was obtained for HRM analysis of *RPB2* amplicons (Figure 5g). For both marker sequences, a gradational distribution of differential melting curves was observed depending on the initial *Sb_745_* cell content of the sample. The smaller the proportion of *Sb_745_* cells in the suspension mix, the further the differential melting curve was from the reference curve. Regarding the adopted clustering parameters, the denaturation curve profiles of Mx_0.5 and S_0.5 products significantly differed from the *Sb*_745_ reference, as shown in the detailed graphs (Figure 5f,h). The melting point of the *HO* product of all the mixtures tested was 79.0 °C, which is consistent with Table S1 data on *HO* T_m_ for *Sc*_D_ and *Sb*_745_ samples. The T_m_ of *RBP2* products amplified with *Sb*_745_ and Mx_Sb DNA templates was 79.8 °C and corresponded to *Sb*_745_ amplicons. *RPB2* amplification products of the other mixed samples achieved T_m_ of 79.4–79.6 °C, in which range the melting point of the *Sc*_D_ sequence falls (Table S1). 

## 4. Discussion

The purpose of this study was to develop a qPCR-HRM-based analysis to identify *Sb* in probiotic-fortified foods. The comparative genomic research demonstrated that *Sb* and *Sc* share more than 99% genomic relatedness as determined by Average Nucleotide identity (ANI) [18]. The aforementioned strong genetic similarity was confirmed herein through performed HRM analysis using the four analyzed regions: *18SrRNA*, *26SrRNA*, ITS and *TEF1alpha*. The generated dendrogram grouped all *Sc* strains with a separate sub-cluster of reference probiotic *Sb* strains. 

Reviewing the melting temperatures of each of the rDNA amplicons obtained in qPCR, there were no significant differences between the *Sc* and *Sb* strains tested. A pair of primers designed for the *18SrRNA* region tested on pure cultures enabled the clustering of subtype *Sb* outside the *Sc* cluster. HRM analysis results concerning *26SrRNA* and ITS sequences resulted in joint clustering of *Sb* and Sc strains. In comparative genomic studies, the sequence ITS1-5.8S rDNA-ITS2 of *Sb* displayed some subtle differences and 99% resemblance. Regarding the sequence of the D1/D2 domain of the *26SrRNA* there was a similarity value of 100% compared to the *Sc* genomes. The probability of constructing an effective differentiation sequence within rDNA is very low, with the scarcity of polymorphisms and the strict requirements for HRM analysis [4,14,22]. The problematic rDNA regions were used in attempts to differentiate species of the genus *Saccharomyces* using HRM analysis. Four primer combinations were designed spanning the *26SrRNA* polymorphic region of 10 *Saccharomyces* species aligned. The highest discrimination level was achieved with five clusters for 10 type strains examined [24]. The combination of *26SrRNA* and ITS regions in another HRM analysis of *Saccharomyces* species enabled discrimination of the most studied yeasts at the species level. However, differentiation of *Sc*, *Sb* and *Saccharomyces uvarum* in the pure cultures or the mixed samples failed [26]. 

Genes encoding actin (*ACT1*), translation elongation factor (*TEF1alpha*), RNA polymerase subunit (*RPB1*) and *COX2*, were considered more effective for the differentiating of the problematic, closely related species [41,42,43]. Moreover, *TEF1alpha* sequencing and MALDI-TOF mass-spectrometry were found more relevant for differentiation within the *Pichia cactophila* clade than sequencing of standard barcoding regions ITS and D1/D2 [44]. Nowadays, *Sc* promoter regions were found not to be fully conserved, in terms of nucleotide sequence nor predicted transcription factor (TF) binding sites, in homolog *Sb* genes. Some of the differentially expressed genes in *Sb* strains were found to have gained or lost TF binding sites in their promoter regions [19]. Therefore, a pair of primers for the yeast polymorphic region within *TEF1alpha* were included in this work. This was effective in interspecies discrimination while showing a very low degree of intraspecies differentiation. Only the *Sc*_EtRed_ strain remained in a distinct cluster from the other *Sc* and *Sb* strains tested. The results revealed that the *Sb* probiotic strains are closer to wine strains of *Sc* than industrial or baking strains [18].

As intended, the primer pairs designed for this work for regions within *CCA1*, *HO*, *RPB2*, *HXT9* and *MAL11* were not amplified in species other than *Sc*. Amplification of *HXT9* and *MAL11* sequences was found only in *Sc* strains. The exception was *Sc*_EtRed_, which showed no *MAL11* amplification. *Sc*_EtRed_ closely relates to wine strains therefore it might show high similarities to *Sb* [45]. MLST (Multilocus Sequence Typing) involved the sequencing of four nuclear genes *CCA1*, *CYT1* (ubiquinol-cytochrome-c reductase catalytic subunit gene), *HMX1* (heme oxygenase gene), *NUP116* (FG-nucleoporin gene) and ITS region resulted in the uniform clade of clinical isolates and commercial probiotic yeasts [23]. In this study, a primers pair designed for the *CCA1* sequence was not sufficient to provide differentiation of probiotic strains. In contrast, the expected effect was found for the *RPB2* sequence. Moreover, HRM analysis with *RPB2* amplicon effectively clustered separately newly isolated *Saccharomyces paradoxus* strain. *HO* sequence clustered *Sb* strains with *Sc*_EtRed_ indicating the close affinity of *Sb* and *Sc* wine strains. 

Comparative analysis with regions in *18S rRNA* and ITS confirmed the presence of *Sc* strains in the dietary supplements tested. Moreover, the selected intraspecies sequences, *HO* and *RPB2*, confirmed the presence of *Sb* in PS2, PS3, PS4, and *Sc* in PS1, according to their composition as declared by the manufacturers. The preparations did not include detailed specifications of the yeast strains declared. As established, *MAL11*, *MAL13* (transcription factor gene), and *ARN2* (siderophore transporter gene) were present in more than 70% of the strains of different subgroups of *Sc* strains but were absent in all the probiotic strains. The large hexose transporter family comprises *HXT11* and *HXT9* which were absent from all strains of *Sb* [18]. Therefore, *HXT9* and *MAL11* sequences were amplified on PS2–PS4 templates. As a result, no amplification products of the *MAL11* region were confirmed, while only for PS2 the *HXT9* amplicon was not detected. Thus, the strains included in the examined supplements, except PS2, do not completely correspond to those with health-promoting properties. 

HRM analysis of DNA extracted from seed mixtures showed reduced sensitivity detection of the selected template compared to the analysis of mixed DNA samples [46]. The yield of bacterial DNA from sourdough fermented with different strains of the same species may differ up to 100,000-fold even if the organisms are present at the same cell counts [47]. The observations support the assumption that the structure of the food matrix could lower the recovery of the nuclear or organellar DNA. It was declared that qPCR-HRM analysis detects but does not identify organisms if they account for 0.1–1% of the bacterial and yeast population, respectively [48]. Therefore, the effectiveness of selected sequences in identifying probiotic strains was tested using DNA templates obtained in extraction from mixtures containing three yeast species. Mixtures were prepared that microbiologically corresponded in composition to kefirs [37,49,50,51,52]. The application of commercial kefirs in the study failed. The reason was the very low yeast content, no more than log 2 CFU mL^−1^ of cells was determined in commercial products. Due to very small differences in melting temperature (T_m_) and melting profile of *18SrRNA* amplicons for *Sb*_745_, *Sc_D_*, *Km* and *Pf*_D_ all mix-yeast samples were clustered together. If the ITS sequence was considered, common clustering of samples containing only strains of *Sc* species was observed (*Sb*_745_ and *Sc*_D_). In contrast, the other samples with additional *Km* and *Pf*_D_ presence formed several separate clusters. ITS region showed the greatest variability in DNA primary structure amongst the analyzed rDNA sequences. Consequently, it has the highest strength of interspecies differentiation in the HRM analysis. Amplification with a pair of ITS primers on DNA extracted from mixtures yielded heterogeneous products as a result of the increased affinity of the primers for the *Pf* sequence, as a consequence of nucleotide changes in the annealing regions of the *Sc* and *Km* sequences [40]. 

The designed intragenus primer pairs for *HO* or *RPB2* regions were the best in the identification of *Sb* in the microbial mixtures. The *RPB2* sequence had the highest differentiation power. The reference probiotic strain was identifiable by qPCR-HRM when the mixture contained at least log 7 CFU mL^−1^ of *Sb* cells, and *Sb*_745_ significantly exceeded the *Sc*_D_ strain quantitatively. Figure 5h shows that the presence of other yeast species does not significantly change the product melting profile for M_Sb. In contrast, a reduction in the number of *Sb*_745_ cells to *Sc*_D_ resulted in a significant increase in difference RFU of 0.2 to the reference. The limits for accurate quantification of *Sc* in wine artificially contaminated, with real-time PCR using specific primers, were established for log 5 CFU mL^−1^ in sweet wine and log 6 CFU mL^−1^ in red wine [53]. Such high detection thresholds by qPCR indicate that the stated threshold for identification of *Sb* cells using qPCR-HRM analysis is plausible. A required minimum dose of *health-boosting microorganisms* is log 6 CFU mL^−1^ or CFU g^−1^ for the food product to be labelled as a probiotic [54]. Since the viability of microorganisms is the key to achieving health benefits (log 6–log 7 CFU per g during the expected shelf-life of the probiotic food or beverage, according to WHO/FAO, 2006, some researchers even suggest increasing the dose up to log 7 CFU mL^−1^ or CFU g^−1^ [55]. This is sufficient enough to detect and identify the probiotic strain of *Sb* yeast in probiotic-enriched foods with qPCR-HRM analysis using the genus specific primer pairs. 

## 5. Conclusions

Preliminary validation of qPCR-HRM analysis using primer pairs’ set and species-diverse yeast samples enabled the selection of interspecies sequences and genus-specific sequences with appropriate differentiation power for *Sb* identification among *Sc* strains. The designed multi-fragment genetic analysis was sufficient for *Sb* identification in pure culture and proved effective in the authentication of dietary supplements’ probiotic composition. 

Successful high-resolution melting analysis highly relies on the purity of the DNA template, but also on the quantity, which must be comparable to the reference template. Consequently, challenges may arise in detecting, and especially in identifying, microorganisms within the microbiologically complex food matrices. In the present study, the identification of *Sb* in the microbial mixtures of kefir succeeded using specific intragenus sequences. Additionally, the established qPCR-HRM analysis enabled the identification of *Sb* in the microbial mix with the predominance of *Sb*, corresponding to the required dose of health-promoting microorganisms for the food product to be labelled as probiotic.

## Figures and Tables

**Table 1:** Composition of tested dietary supplements.

LABEL	COMPOSITION
PS1	*Saccharomyces cerevisiae*
PS2	*Lactiplantibacillus plantarum*, *Bifidobacterium breve*, *Saccharomyces boulardii*
PS3	*Saccharomyces boulardii* and fructooligosaccharides
PS4	*Saccharomyces boulardii*, *L. rhamnosus* GG, fructooligosaccharides

**Table 2:** Description of the primer pairs designed to amplify up to 250 bp regions within seven genes. Accession numbers correspond to the sequences of *Saccharomyces cerevisiae* S288C.

GENE			PRIMERS				
NAME (SYMBOL)	ACCESSION NUMBER	RANGE [bp]	LABEL	START	STOP	SEQUENCE (5′->3′)	PRODUCT LENGTH [bp]
Homothallic switching endonuclease (*HO*)	NC_001136.1	46,810–47,130	HO_Fw	46,817	46,836	TGAAGTTGTTCCCCCAGCAA	198
			HO_Rv	47,014	46,995	GGCGAAGGCCCTGAATCTTA	
DNA-directed RNA polymerase II core subunit (*RPB2*)	NC_001147.6	615,169–615,510	RPB2_Fw	615,217	615,239	ACGGTTCAAAACCTGAGAAACAC	229
			RPB2_Rv	615,445	615,424	AGGTCCATTATTGGCCCAACTT	
tRNA adenylyl transferase (*CCA1*)	NC_001137.3	522,230–522,503	CCA1_Fw	522,282	522,302	CCAGATGCTTGGATTTCTCGG	213
			CCA1_Rv	522,494	522,474	AGCCATTGACTCTTCGGATCA	
Hexose transporter (*HXT9*)	NC_001142.9	19,800–20,028	HXT9_Fw	19,824	19,846	AGAATGGGTTTGATCGTCTCAAT	197
			HXT9_Rv	20,020	19,996	AGGCCAGAAATAATTCTTCCAATGA	
Alpha-glucosidase permease (*MAL11*)	NC_001139.9	1,074,890–1,075,090	MAL11_Fw	1,074,891	1,074,910	TTTCTCACCAACCACCAGGG	196
			MAL11_Rv	1,075,086	1,075,065	ACATGACCAGTTACTCCAACAT	
Topoisomerase I damage affected (*TDA8*)	NC_001133.9	13,363–13,743	TDA8_Fw	13,380	13,399	GGGCTGTTAGGTCATCGTCA	182
			TDA8_Rv	13,561	13,542	GCCCGATAACATTGCAGGGA	
Translation elongation factor 1-alpha (*TEF1alpha*)	NC_001142.4	701,120–701,420	TEF1_Fw	701,138	701,159	ACCCAAAGACTGTTCCATTCGT	238
			TEF1_Rv	701,375	701,355	GGCACAGTACCAATACCACCA	

## Data Availability

The original contributions presented in the study are included in the article/Supplementary Material, further inquiries can be directed to the corresponding author/s.

## Supplementary Materials

The following supporting information can be downloaded at: https://www.mdpi.com/article/10.3390/biotech13040048/s1, Figure S1: Microbial mixtures’ quantitative composition; Figure S2: Amplicons’ length verification; Figure S3: Dendrogram illustrating the similarity level of all studied yeast strains; Table S1: The melting temperature (Tm) [°C] analysis results for all studied targets and sequences.

## References

[1] Hill C., Guarner F., Reid G., Gibson G.R., Merenstein D.J., Pot B., Morelli L., Canani R.B., Flint H.J., Salminen S. (2014). Expert Consensus Document: The International Scientific Association for Probiotics and Prebiotics Consensus Statement on the Scope and Appropriate Use of the Term Probiotic. Nat. Rev. Gastroenterol. Hepatol..

[2] Fijan S. (2014). Microorganisms with Claimed Probiotic Properties: An Overview of Recent Literature. Int. J. Environ. Res. Public Health.

[3] Łukaszewicz M. (2012). Saccharomyces Cerevisiae Var. Boulardii—Probiotic Yeast. Probiotics.

[4] Fietto J.L.R., Araújo R.S., Valadão F.N., Fietto L.G., Brandão R.L., Neves M.J., Gomes F.C.O., Nicoli J.R., Castro I.M. (2004). Molecular and Physiological Comparisons between *Saccharomyces cerevisiae* and *Boulardii*. Can. J. Microbiol..

[5] Czerucka D., Piche T., Rampal P. (2007). Review article: Yeast as Probiotics—*Saccharomyces boulardii*. Aliment. Pharmacol. Ther..

[6] Cesaro S., Chinello P., Rossi L., Zanesco L. (2000). *Saccharomyces cerevisiae* Fungemia in a Neutropenic Patient Treated with *Saccharomyces boulardii*. Support. Care Cancer.

[7] Thygesen J.B., Glerup H., Tarp B. (2012). *Saccharomyces boulardii* Fungemia Caused by Treatment with a Probioticum. BMJ Case Reports.

[8] Santino L., Alari A., Bono S., Tetp E., Bernardini A., Magrini L., Di Somma S., Teggi A. (2014). *Saccharomyces cerevisiae* fungemia, a possible consequence of the treatment of *Clostridium difficile* colitis with a probioticum. Int. J. Immunopathol. Pharmacol..

[9] Ellouze O., Berthoud V., Mervant M., Parthiot J.P., Girard C. (2016). Septic Shock Due to *Saccharomyces boulardii*. Med. Et Mal. Infect..

[10] Kara I., Yıldırım F., Özgen Ö., Erganiş S., Aydoğdu M., Dizbay M., Gürsel G., Kalkanci A. (2018). *Saccharomyces cerevisiae* Fungemia after Probiotic Treatment in an Intensive Care Unit Patient. J. Mycol. Med..

[11] Cardinali G., Martini A. (1994). Electrophoretic Karyotypes of Authentic Strains of the Sensu Strict0 Group of the Genus *Saccharomyces*?. Int. J. Syst. Bacteriol..

[12] Molnar O., Messner R., Prillinger H., Stahl U., Slavikova E. (1995). Genotypic Identification of *Saccharomyces* Species Using Random Amplified Polymorphic DNA Analysis. Syst. Appl. Microbiol..

[13] McCullough M.J., Clemons K.V., McCusker J.H., Stevens D.A. (1998). Species Identification and Virulence Attributes of *Saccharomyces boulardii* (Nom. Inval.). J. Clin. Microbiol..

[14] Van Der Aa Kühle A., Jespersen L. (2003). The Taxonomic Position of *Saccharomyces boulardii* as Evaluated by Sequence Analysis of the D1/D2 Domain of 26S RDNA, the ITS1-5.8S RDNA-ITS2 Region and the Mitochondrial Cytochrome-c Oxidase II Gene. Syst. Appl. Microbiol..

[15] Kurtzman C.P., Robnett C.J. (1998). Identification and Phylogeny of Ascomycetous Yeasts from Analysis of Nuclear Large Subunit (26S) Ribosomal DNA Partial Sequences. Antonie Van Leeuwenhoek.

[16] McFarland L.V. (1996). *Saccharomyces boulardii* is not *Saccharomyces cerevisiae*. Clin. Infect. Dis..

[17] Edwards-Ingram L., Gitsham P., Burton N., Warhurst G., Clarke I., Hoyle D., Oliver S.G., Stateva L. (2007). Genotypic and Physiological Characterization of *Saccharomyces boulardii*, the Probiotic Strain of *Saccharomyces cerevisiae*. Appl. Environ. Microbiol..

[18] Khatri I., Tomar R., Ganesan K., Prasad G.S., Subramanian S. (2017). Complete Genome Sequence and Comparative Genomics of the Probiotic Yeast *Saccharomyces boulardii*. Sci. Rep..

[19] Pais P., Oliveira J., Almeida V., Yilmaz M., Monteiro P.T., Teixeira M.C. (2021). Transcriptome-Wide Differences between *Saccharomyces cerevisiae* and *Saccharomyces cerevisiae* Var. *Boulardii*: Clues on Host Survival and Probiotic Activity Based on Promoter Sequence Variability. Genomics.

[20] Mitterdorfer G., Mayer H.K., Kneifel W., Viernstein H. (2002). Clustering of *Saccharomyces boulardii* Strains within the Species *S. Cerevisiae* Using Mol. Typing Techniques. J. Appl. Microbiol..

[21] Hennequin C., Thierry A., Richard G.F., Lecointre G., Nguyen H.V., Gaillardin C., Dujon B. (2001). Microsatellite Typing as a New Tool for Identification of *Saccharomyces cerevisiae* Strains. J. Clin. Microbiol..

[22] Posteraro B., Sanguinetti M., Romano L., Torelli R., Novarese L., Fadda G. (2005). Molecular Tools for Differentiating Probiotic and Clinical Strains of *Saccharomyces cerevisiae*. Int. J. Food Microbiol..

[23] Imre A., Rácz H.V., Antunovics Z., Rádai Z., Kovács R., Lopandic K., Pócsi I., Pfliegler W.P. (2019). A New, Rapid Multiplex PCR Method Identifies Frequent Probiotic Origin among Clinical *Saccharomyces* Isolates. Microbiol. Res..

[24] Nadai C., Bovo B., Giacomini A., Corich V. (2018). New Rapid PCR Protocol Based on High-Resolution Melting Analysis to Identify *Saccharomyces cerevisiae* and Other Species within Its Genus. J. Appl. Microbiol..

[25] Ripari V., Gänzle M.G., Berardi E. (2016). Evolution of Sourdough Microbiota in Spontaneous Sourdoughs Started with Different Plant Materials. Int. J. Food Microbiol..

[26] Bazalová O., Cihlář J.Z., Dlouhá Z., Bár L., Dráb V., Kavková M. (2022). Rapid Sourdough Yeast Identification Using Panfungal PCR Combined with High Resolution Melting Analysis. J. Microbiol. Methods.

[27] Erdem M., Kesmen Z., Özbekar E., Çetin B., Yetim H. (2016). Application of High-Resolution Melting Analysis for Differentiation of Spoilage Yeasts. J. Microbiol..

[28] Kesmen Z., Büyükkiraz M.E., Özbekar E., Çelik M., Özkök F.Ö., Kılıç Ö., Çetin B., Yetim H. (2018). Assessment of Multi Fragment Melting Analysis System (MFMAS) for the Identification of Food-Borne Yeasts. Curr. Microbiol..

[29] Kesmen Z., Özbekar E., Büyükkiraz M.E. (2018). Multifragment Melting Analysis of Yeast Species Isolated from Spoiled Fruits. J. Appl. Microbiol..

[30] Baleiras-Couto M.M., Eijsma B., Hofstra H., Huis In’T Veld J.H., Van Der Vossen J.M. (1996). Evaluation of Molecular Typing Techniques to Assign Genetic Diversity among *Saccharomyces cerevisiae* Strains. Appl. Environ. Microbiol..

[31] Valente P., Gouveia C., De Lemos G.A., Pimentel D., Van Elsas J.D., Mendonqa-Hagler L.C., Hagler A.N. (1996). PCR Amplification of the RDNA Internal Transcribed Spacer Region for Differentiation of *Saccharomyces* Cultures. FEMS Microbiol. Lett..

[32] Rekha C.R., Vijayalakshmi G. (2010). Bioconversion of Isoflavone Glycosides to Aglycones, Mineral Bioavailability and Vitamin B Complex in Fermented Soymilk by Probiotic Bacteria and Yeast. J. Appl. Microbiol..

[33] Değirmencioğlu N., Gurbuz O., Şahan Y. (2016). The Monitoring, Via an In Vitro Digestion System, of the Bioactive Content of Vegetable Juice Fermented with *Saccharomyces cerevisiae* and *Saccharomyces boulardii*. J. Food Process. Preserv..

[34] Zamora-Vega R., Montañez-Soto J.L., Martínez-Flores H.E., Flores-Magallón R., Muñoz-Ruiz C.V., Venegas-González J., De Jesús Ariza Ortega T. (2012). Effect of Incorporating Prebiotics in Coating Materials for the Microencapsulation of *Sacharomyces boulardii*. Int. J. Food Sci. Nutr..

[35] Chan M.Z.A., Tan L.T., Heng S.W.Q., Liu S.Q. (2023). Effect of Co-Fermentation of *Saccharomyces boulardii* CNCM-I745 with Four Different Probiotic *Lactobacilli* in Coffee Brews on Cell Viabilities and Metabolic Activities. Fermentation.

[36] Karaolis C., Botsaris G., Pantelides I., Tsaltas D. (2013). Potential Application of *Saccharomyces boulardii* as a Probiotic in Goat’s Yoghurt: Survival and Organoleptic Effects. Int. J. Food Sci. Technol..

[37] Goktas H., Dikmen H., Demirbas F., Sagdic O., Dertli E. (2021). Characterisation of Probiotic Properties of Yeast Strains Isolated from Kefir Samples. Int. J. Dairy Technol..

[38] Gut A.M., Vasiljevic T., Yeager T., Donkor O.N. (2019). Characterization of Yeasts Isolated from Traditional Kefir Grains for Potential Probiotic Properties. J. Funct. Foods.

[39] Sambrook J., Russell D. (2001). Molecular Cloning: A Laboratory Manual.

[40] Borkowska M., Celińska E. (2023). Multiple Region High Resolution Melting-Based Method for Accurate Differentiation of Food-Derived Yeasts at Species Level Resolution. Food Microbiol..

[41] Cappello M.S., Poltronieri P., Blaiotta G., Zacheo G. (2010). Molecular and Physiological Characteristics of a Grape Yeast Strain Containing Atypical Genetic Material. Int. J. Food Microbiol..

[42] Eizaguirre J.I., Peris D., Rodríguez M.E., Lopes C.A., De Los Ríos P., Hittinger C.T., Libkind D. (2018). Phylogeography of the Wild Lager-Brewing Ancestor (*Saccharomyces eubayanus*) in Patagonia. Environ. Microbiol..

[43] Hulin M., Harrison E., Stratford M., Wheals A.E. (2014). Rapid Identification of the Genus *Dekkera/Brettanomyces*, the *Dekkera* Subgroup and All Individual Species. Int. J. Food Microbiol..

[44] Guitard J., Atanasova R., Brossas J.Y., Meyer I., Gits M., Marinach C., Vellaissamy S., Angoulvant A., Mazier D., Hennequin C. (2015). Candida Inconspicua and Candida Norvegensis: New Insights into Identification in Relation to Sexual Reproduction and Genome Organization. J. Clin. Microbiol..

[45] Gronchi N., De Bernardini N., Cripwell R.A., Treu L., Campanaro S., Basaglia M., Foulquié-Moreno M.R., Thevelein J.M., Van Zyl W.H., Favaro L. (2022). Natural *Saccharomyces cerevisiae* Strain Reveals Peculiar Genomic Traits for Starch-to-Bioethanol Production: The Design of an Amylolytic Consolidated Bioprocessing Yeast. Front. Microbiol..

[46] Emenyeonu L.C., Croxford A.E., Wilkinson M.J. (2018). The Potential of Aerosol EDNA Sampling for the Characterisation of Commercial Seed Lots. PLoS ONE.

[47] Zheng J., Ruan L., Sun M., Gänzle M. (2015). A Genomic View of *Lactobacilli* and *Pediococci* Demonstrates That Phylogeny Matches Ecology and Physiology. Appl. Environ. Microbiol..

[48] Lin X.B., Gänzle M.G. (2014). Quantitative High-Resolution Melting PCR Analysis for Monitoring of Fermentation Microbiota in Sourdough. Int. J. Food Microbiol..

[49] Wang S.Y., Chen H.C., Liu J.R., Lin Y.C., Chen M.J. (2008). Identification of Yeasts and Evaluation of Their Distribution in Taiwanese Kefir and Viili Starters. J. Dairy Sci..

[50] Kalamaki M.S., Angelidis A.S. (2017). Isolation and Molecular Identification of Yeasts in Greek Kefir. Int. J. Dairy Technol..

[51] Özer B., Kirmaci H.A. (2014). Fermented Milks: Products of Eastern Europe and Asia. Encyclopedia of Food Microbiology.

[52] Nejati F., Junne S., Kurreck J., Neubauer P. (2020). Quantification of Major Bacteria and Yeast Species in Kefir Consortia by Multiplex TaqMan QPCR. Front. Microbiol..

[53] Martorell P., Querol A., Fernández-Espinar M.T. (2005). Rapid Identification and Enumeration of *Saccharomyces cerevisiae* Cells in Wine by Real-Time PCR. Appl. Environ. Microbiol..

[54] White J., Hekmat S. (2018). Development of Probiotic Fruit Juices Using *Lactobacillus rhamnosus* GR-1 Fortified with Short Chain and Long Chain Inulin Fiber. Fermentation.

[55] Fiocco D., Longo A., Arena M.P., Russo P., Spano G., Capozzi V. (2020). How Probiotics Face Food Stress: They Get by with a Little Help. Crit. Rev. Food Sci. Nutr..

