# Supplementary material for: High-Resolution Melting Analysis Potential for Saccharomyces cerevisiae var. boulardii Authentication in Probiotic-Enriched Food Matrices
Source: BioTech (Basel). 2024 Nov 14;13(4):48. doi: 10.3390/biotech13040048 (PMC11586983; doi:10.3390/biotech13040048)
Supplement: Supplementary file 1 [file biotech-13-00048-s001.zip › biotech-3217394-supplementary.pdf]

Supplementary Materials: High-Resolution Melting Analysis Potential for *Saccharomyces cerevisiae* var. *boulardii* Authentication in Probiotic-Enriched Food Matrices

Monika Borkowska, Michał Kułakowski and Kamila Myszka

a

Samples:	*Cells per isolation*					*Cells in total*	*Yeast cells in total*	*DNA [ng µL⁻¹]*	*DNA per qPCR reaction [ng]*
	*Ld*subl	Sc_D	Sb_{745}	*Km*	Pf_D				
Mx_*Sc*	2,2E+08	1,2E+07	0,0E+00	7,6E+06	4,0E+05	2,4E+08	2,0E+07	20,7	20
Mx_0.1 [S_0.1]	2,2E+08 [-]	1,1E+07	1,2E+06	7,6E+06 [-]	4,0E+05 [-]	2,4E+08	2,0E+07	14,9 [15,4]	20
Mx_0.5 [S_0.5]	2,2E+08 [-]	6,0E+06	6,0E+06	7,6E+06 [-]	4,0E+05 [-]	2,4E+08	2,0E+07	14 [24,1]	20
Mx_0.9 [S_0.9]	2,2E+08 [-]	1,2E+06	1,1E+07	7,6E+06 [-]	4,0E+05 [-]	2,4E+08	2,0E+07	13,3 [12,3]	20
Mx_*Sb*	2,2E+08	0,0E+00	1,2E+07	7,6E+06	4,0E+05	2,4E+08	2,0E+07	22,3	20

b

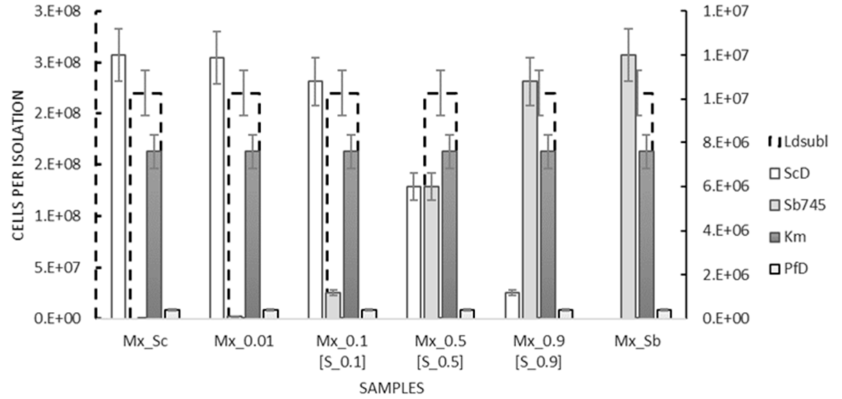

Figure S1. Microbial mixtures' quantitative composition. Numerical (a) and graphical (b) presentation. Table a details DNA concentration after pellets' extraction and effective DNA quantity used in qPCR reactions. *Ldsubl*—*Lactobacillus delbrueckii* subsp. *lactis* DSM 20072, Sc_D—isolate *S. cerevisiae* from DBFM collection, Sb_{745} —reference strain *S. cerevisiae* var. *boulardii* CNCM I-745, *Km*—*K. marxianus* DSM 5422, Pf_D—food-derived isolate *P. fermentans* from DBFM collection, Mx_*Sb* ratio—multi-species yeast mixture, S_ *Sb* ratio—*S. cerevisiae* mixture.

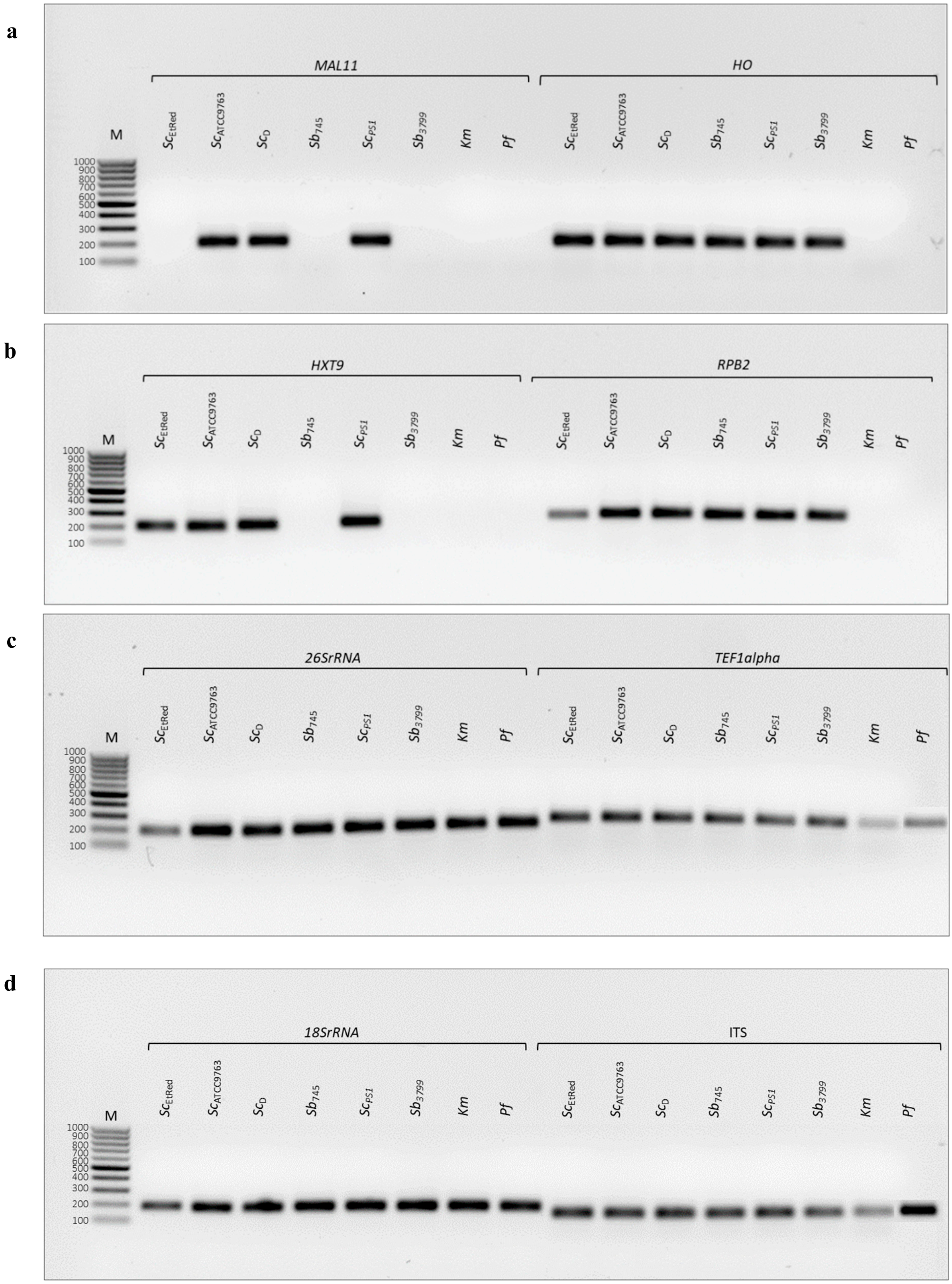
a
MAL11
HO
M
1000
900
800
700
600
500
400
300
200
100
Sc EtRed
Sc ATCC9763
Sc D
Sb 745
Sc PS1
Sb 3799
Km
Pf
b
HXT9
RPB2
c
26SrRNA
TEF1alpha
d
18SrRNA
ITS

e

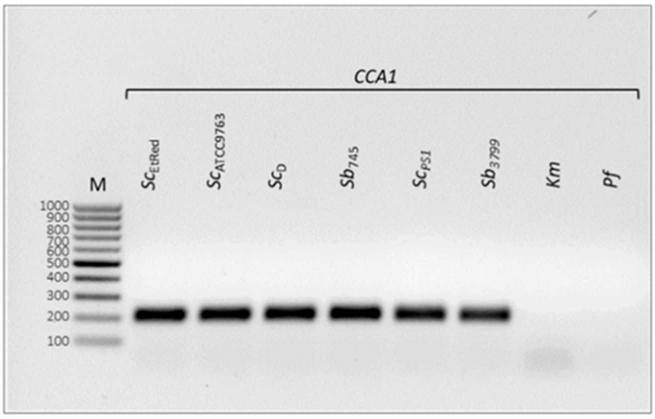

Figure S2. Amplicons' length verification. Electrophoretic separation of *MAL11* and *HO* (**a**), *HXT9* and *RPB2* (**b**), *18S rDNA* and ITS (c), *26S rDNA* and *TEF1alpha* (d) and *CCA1* (e) amplicons obtained in PCR for *S. cerevisiae* var. *boulardii* reference strains (Sb_{745}, Sb_{3799}), *S. cerevisiae* strains ($Sc_{ATCC9763}$, Sc_{EtRed}, Sc_{D} and Sc_{PS1}), *K. marxianus* (*Km*) and *P. fermentans* (*Pf*). M—DNA Marker 100bp LOAD (Syngen Biotech, Wroclaw, Poland).

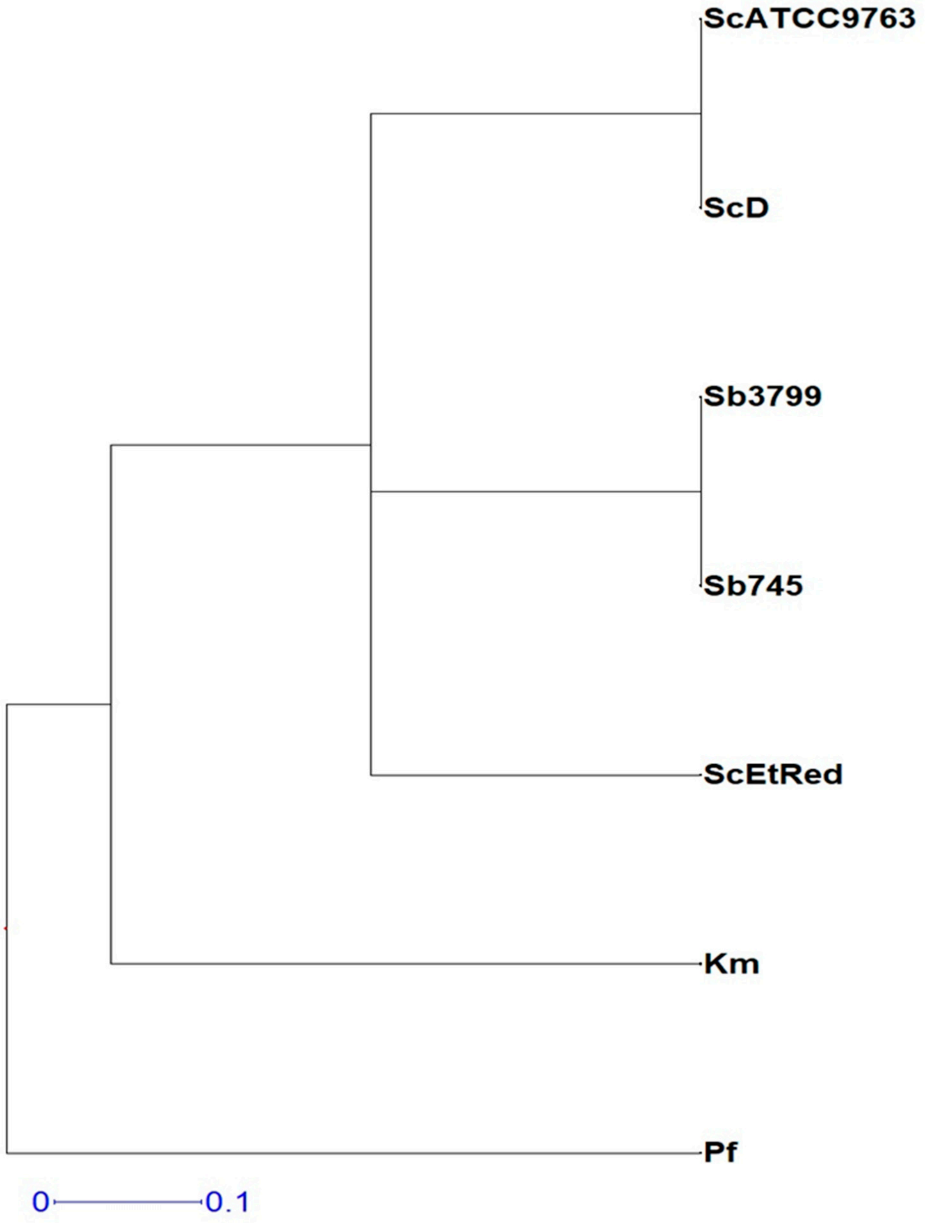

Figure S3. Dendrogram illustrating the similarity level of all studied yeast strains. Based on clustering in HRM analysis of four targeted regions (*18SrRNA*, *26SrRNA*, ITS and *TEF1alpha*). Diversity analysis was conducted using DarWin version 6.0.21 software. The strains were grouped using the unweighted pair-group method with the arithmetic averages (UPGMA) clustering algorithm.

Table S1. The melting temperature (Tm) [°C] analysis results for all studied targets and sequences. Tm1 and Tm2 are technical repeats of qPCR-HRM analysis readouts.

**TARGET**	***18SrRNA***		**ITS**		***26SrRNA***		***TEF1alpha***		***CCA1***		***HO***		***RPB2***		***MAL11***		***HXT9***	
	**Tm_1**	**Tm_2**	**Tm_1**	**Tm_2**	**Tm_1**	**Tm_2**	**Tm_1**	**Tm_2**	**Tm_1**	**Tm_2**	**Tm_1**	**Tm_2**	**Tm_1**	**Tm_2**	**Tm_1**	**Tm_2**	**Tm_1**	**Tm_2**
$Sc_{ATCC9763}$	83,8	83,8	82,2	82,4	79,8	79,8	82,8	82,6	81,8	81,8	79,0	79,2	79,6	79,6	79,8	79,8	79,2	79,2
Sc_{D}	83,8	83,8	82,2	82,4	79,8	79,8	82,6	82,6	82,0	82,0	79,0	79,0	79,6	79,6	79,8	79,8	79,0	79,0
Sc_{EtRed}	83,8	83,8	82,2	82,4	79,8	79,8	82,6	82,4	82,4	82,4	79,0	79,0	79,6	79,6	None	None	79,2	79,2
Sb_{745}	83,8	83,8	82,2	82,4	79,8	79,8	82,6	82,6	82,0	82,0	79,0	79,0	79,8	79,8	None	None	None	None
Sb_{3799}	83,8	83,8	82,2	82,2	79,6	79,6	82,6	82,6	82,2	82,2	79,0	79,0	79,8	79,8	None	None	None	None
Km	84,0	84,0	81,6	81,6	79,6	79,6	83,0	83,0	None	None	None	None	None	None	None	None	None	None
Pf	84,4	84,4	84,4	84,6	81,0	81,0	81,8	81,8	None	None	None	None	None	None	None	None	None	None
